# Multiple infected lung bullae associated with *Mycobacterium intracellulare* in a Japanese man

**DOI:** 10.1002/rcr2.734

**Published:** 2021-03-08

**Authors:** Tatsuya Kodama, Atsushi Kurokawa, Hiroyuki Kokuto

**Affiliations:** ^1^ Department of Respiratory Medicine Self‐Defense Forces Central Hospital Tokyo Japan; ^2^ Department of Respiratory Medicine Fukujuji Hospital, Japan Anti‐Tuberculosis Association Kiyose Japan

**Keywords:** Infected lung bulla, *Mycobacterium intracellulare*, tetralogy of Fallot

## Abstract

*Mycobacterium avium* complex (MAC)‐infected lung bulla was a rare type of pulmonary non‐tuberculous mycobacterial (NTM) infection. A 29‐year‐old man with a history of tetralogy of Fallot was admitted to our hospital because of a high fever and left chest pain. Chest computed tomography showed two bullae with intrabullous fluid in both the lower lobes and centrilobular small nodular shadow in the right upper lobe and the left lower lobe. Culture of bronchoscopic washing specimen from the right upper lobe bronchus and left lower lobe one and purulent fluid drained from the bulla in the left lower lobe revealed *Mycobacterium intracellulare*. Percutaneous drainage from the left bulla and anti‐NTM treatment were performed. Afterwards, symptoms improved and two intrabullous fluid disappeared. Therefore, a diagnosis of multiple infected lung bullae associated with *M. intracellulare* was made. This is the first documented case of multiple infected lung bullae associated with MAC.

## Graphical abstract

Here, we report the first documented case of multiple infected lung bullae associated with *Mycobacterium avium* complex (MAC) in a 29‐year‐old man.

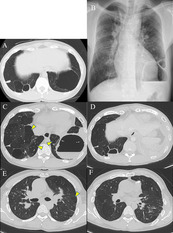

## Introduction

*Mycobacterium intracellulare* and *Mycobacterium avium* as part of *M*. *avium* complex (MAC) are the most common pathogens causing pulmonary infection among non‐tuberculous mycobacteria (NTM) in Japan. Pulmonary infection caused by MAC are mainly categorized into three types: the tuberculosis type, the small nodule or bronchial lesion type, and the disseminated type [1]. MAC‐infected lung bulla was a rare type of pulmonary NTM infection. We experienced a case of multiple infected lung bullae associated with *M. intracellulare*. Although there have been several reports related to infected lung bulla associated with MAC [2, 3], on literature search, this is the first case of multiple infected lung bullae associated with MAC.

## Case Report

**Figure 1:**
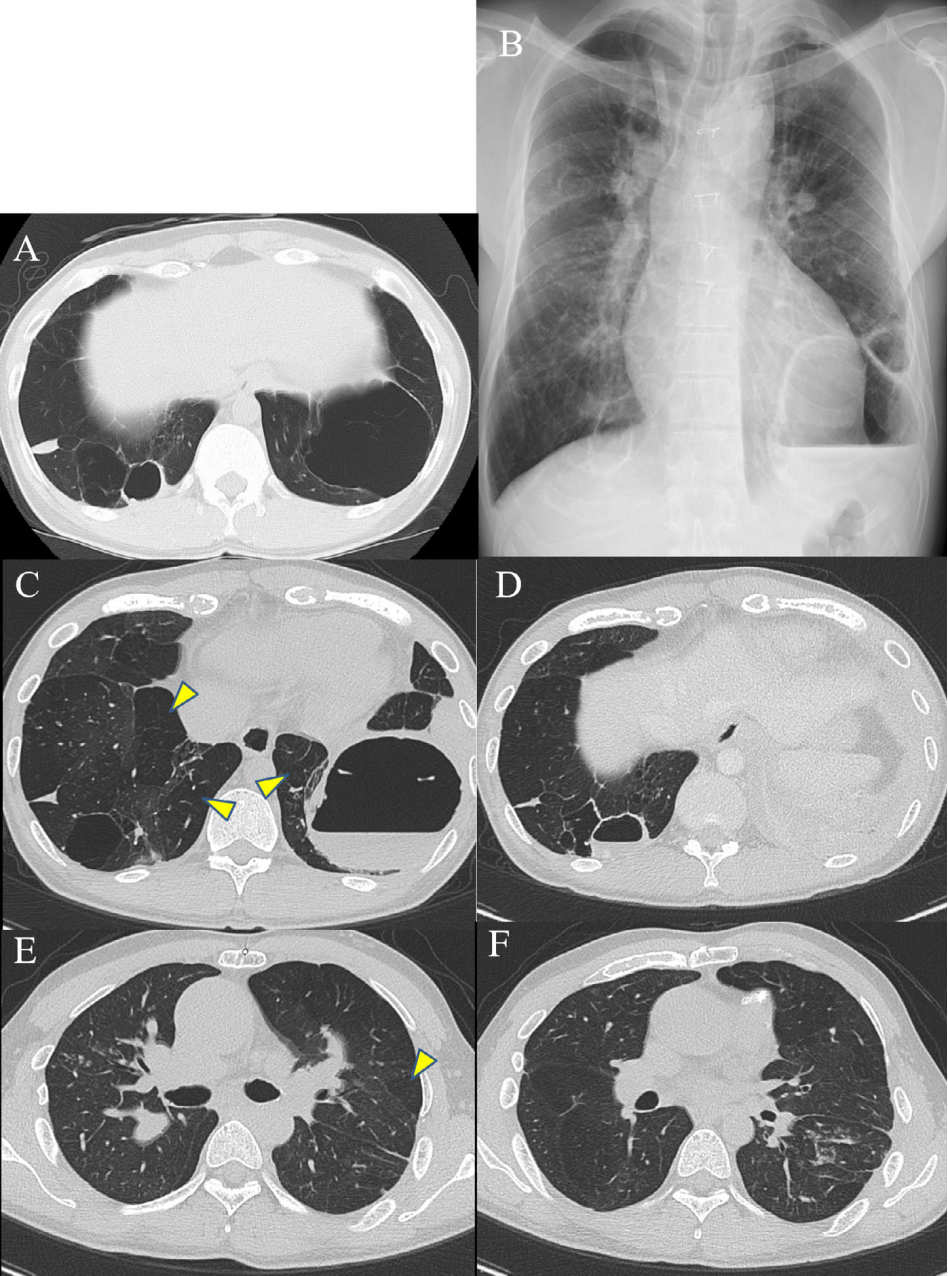
(A) Chest computed tomography (CT) performed one year before hospitalization showed the lung bullae without fluid in both the lower lung lobes. (B) Chest X‐ray on admission showed marked bullous lesions in both the lower lung fields. There was a bulla with an air‐fluid level in the left lower lung field. (C–F) Chest CT on admission showed a bulla (C: 7 × 7 cm) with intrabullous fluid and infiltration shadow surrounding the bulla in the left lower lobe, and a bulla (D: 3 × 2 cm) with a thickened wall and an intrabullous fluid in the right lower lobe. It also showed trans‐airway scattered small nodular nodules in the right S^2^ and left S^8^. There were low attenuation areas in both the lung lobes (arrowheads).

**Figure 2:**
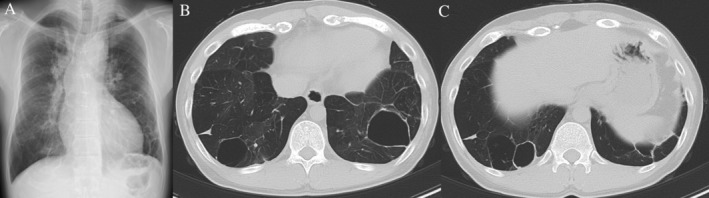
(A) Chest X‐ray at the end of treatment showed that the air‐fluid level in the left lung bulla disappeared. (B, C) Chest computed tomography (CT) at the end of treatment showed that the intrabullous fluid in both the lungs disappeared and left bulla decreased in size. Wall thickness of both bullae reduced.

A 29‐year‐old man was admitted to hospital with a high fever and left chest pain. He was born with a low birth weight and had a history of post‐natal oxygen therapy. In addition to this, he had a history of tetralogy of Fallot (TOF) with pulmonary valve atresia and major aortopulmonary collateral arteries and underwent several operations in early childhood to address these anomalies. He was not immunocompromised and was a non‐smoker. Multiple bullae and low attenuation area in both lungs had been pointed out since early childhood. Chest computed tomography (CT) performed a year before admission showed the lung bullae without intrabullous fluid in both the lower lung lobes (Fig. 1A). On admission, laboratory tests showed increased inflammatory markers (white blood cells count: 11,640/μL, C‐reactive protein: 12.3 mg/dL). Chest radiography revealed intrabullous fluid in the lower part of the left lung (Fig. 1B). Chest CT showed two bullae with intrabullous fluid in both the lower lobes and centrilobular small nodular shadow in the right upper lobe and left lower one, and there was low attenuation area in both the lung lobes (Fig. 1C–F). The patient's symptoms and intrabullous fluid did not improve despite treatment with ampicillin/sulbactam. Culture of bronchial washing specimen from the right upper lobe bronchus and left lower lobe one was negative for bacteria but positive for acid‐fast bacilli. Polymerase chain reaction and culture revealed *M. intracellulare*. Percutaneous drainage from the bulla with intrabullous fluid on the left lower lobe was performed and a drainage tube was inserted. Purulent fluid was drained and found to contain mainly lymphocytes. Findings on culture of this fluid were the same as those of the bronchial washing specimen. There were no fungal isolates. Therefore, we diagnosed infected left lung bulla associated with *M. intracellulare*. He was treated with rifampicin, clarithromycin, ethambutol, and intermittent amikacin in accordance with the American Thoracic Society/Infectious Diseases Society of America recommendations for severe MAC lung disease [4]. The drainage tube on the left side was removed when fluid was no longer draining. His fever and chest pain gradually improved. Inflammatory markers also decreased to normal range. Intermittent amikacin therapy was completed in three months, and other triple therapy was continued for two years. During the course of treatment, we confirmed the negative finding of sputum for *M. intracellulare* four months after starting treatment. Imaging at the end of the treatment revealed that the intrabullous fluid in both the lung bullae disappeared and wall thickness of both bullae reduced (Fig. 2A, B). Based on these findings, a diagnosis of multiple infected lung bullae associated with *M. intracellulare* was made. In addition to this, small nodular shadow in the right middle lobe and left lower lobe also disappeared. There has been no recurrence to date.

## Discussion

Infected lung bulla associated with MAC is very rare and no cases of multiple bullae have been reported.

According to several reports of infected lung bulla associated with MAC, they were diagnosed by resected lung specimen [2, 3]. In our case, we performed percutaneous drainage to examine intrabullous fluid in the left lung bulla as we considered his history of several surgeries for TOF and extensive intrapleural adhesion was estimated based on the surgical diagnosis and treatment. On considering the percutaneous drainage, we need to be aware of risks of pneumothorax, haemothorax, and empyema.

From the case reports that performed surgical treatment and diagnosed as infectious lung bullae caused by MAC, it was suggested that there was no communication between bulla and respiratory tract in the pathological findings [2, 3]. In our case, there were small nodular lesions and infected lung bullae lesions in both lungs. We could not tell how those two lesions were related as we did not perform surgical resection and include pathological findings.

In our case, existence of bullae had been pointed since childhood and chest CT had shown lung bullae without fluid in both the lower lobes one year before admission (Fig. 1A). It is more plausible that MAC infected the lung bulla walls originally, and the appearance of intrabullous fluid and the spread of inflammation to the surrounding tissue occurred as the disease progressed than that the cavitation with intracavitary fluid was formed following the MAC infection in lung parenchyma.

The aetiology of this patient's pre‐existing lung bullae is unclear. However, one possible cause was bronchopulmonary dysplasia due to his low birth weight and history of post‐natal oxygen therapy considering the fact that multiple bullae and low attenuation area had been pointed out since early childhood [4].

Our experience with this patient suggests that concomitant use of percutaneous drainage and anti‐NTM therapy should be considered. We treated the patient for two years according to the previously described recommendations [5]. However, there is no fixed view of the treatment period for infected lung bulla associated with MAC. Patients should be carefully monitored for recurrence.

### Disclosure Statement

Appropriate written informed consent was obtained for publication of this case report and accompanying images.
